# *CYP2D6* Phenotypes and Emergency Department Visits Among Patients Receiving Opioid Treatment

**DOI:** 10.1001/jamanetworkopen.2025.23543

**Published:** 2025-07-28

**Authors:** Noor A. Nahid, Caitrin W. McDonough, Yu-Jung Jenny Wei, Yan Gong, Philip E. Empey, Andrew Haddad, Roger B. Fillingim, Julie A. Johnson

**Affiliations:** 1 Department of Pharmacotherapy and Translational Research, University of Florida, Gainesville; 2 Center for Pharmacogenomics and Precision Medicine, University of Florida College of Pharmacy, Gainesville; 3 Department of Biomedical Informatics, The Ohio State University College of Medicine, Columbus; 4 Division of Pharmaceutics and Pharmacology, College of Pharmacy, The Ohio State University, Columbus; 5 Department of Pharmacy and Therapeutics, University of Pittsburgh School of Pharmacy, Pittsburgh, Pennsylvania; 6 Department of Community Dentistry and Behavioral Science, University of Florida, Gainesville; 7 Pain Research and Intervention Center of Excellence, University of Florida, Gainesville; 8 Clinical and Translational Science Institute, The Ohio State University, Columbus; 9 Department of Internal Medicine, The Ohio State University College of Medicine, Columbus; 10 Department of Pharmaceutics & Pharmacology, The Ohio State University College of Pharmacy, Columbus

## Abstract

**Importance:**

Cytochrome P450 2D6 (*CYP2D6*) bioactivates hydrocodone, tramadol, codeine, and oxycodone to active metabolites that primarily provide analgesic activity. Reduced *CYP2D6* activity may be associated with poor pain control.

**Objective:**

To evaluate associations of impaired *CYP2D6* activity based on genotype or *CYP2D6* inhibitors, alone and together, with analgesic activity of *CYP2D6*-metabolized opioids among patients with pain.

**Design, Setting, and Participants:**

This retrospective national, community-based cohort study used electronic health records and genetics data from the All of Us Research Program. Participants included adults prescribed at least 1 *CYP2D6*-metabolized opioid for more than 7 days between January 1, 2014, and December 31, 2022, with whole-genome sequencing data available. Analysis groups were defined by *CYP2D6* phenotype, which was determined based on *CYP2D6* genotype or *CYP2D6* inhibitor–mediated phenoconversion. Statistical analysis was performed from July 1, 2023, to January 15, 2025.

**Exposures:**

*CYP2D6*-metabolized opioids, with or without concomitant *CYP2D6* inhibitor exposure, based on prescription records and overlap with opioids.

**Main Outcomes and Measures:**

The primary outcome was occurrence of any pain-related emergency department (ED) visits during opioid treatment, up to 60 days after opioid initiation. The association between ED visits and *CYP2D6* phenotype was assessed using inverse probability treatment weighting–adjusted logistic regression. Additional analyses were conducted by drug and isolating *CYP2D6* genotype and inhibitors.

**Results:**

Among 31 669 patients (mean [SD] age, 51.2 [15.4] years; 66.5% women) prescribed *CYP2D6*-metabolized opioids, 15 960 had reduced *CYP2D6* activity, and 15 709 had normal or high *CYP2D6* activity based on genotype and inhibitors. A higher percentage of patients with reduced *CYP2D6* activity (hereafter referred to as phenotypic intermediate metabolizers [pIMs] or phenotypic poor metabolizers [pPMs]) had experienced pain-related ED visits compared with patients with normal or high *CYP2D6* activity (phenotypic normal metabolizers [pNMs] and phenotypic ultrarapid metabolizers [pUMs]) (2.1% vs 1.8%; inverse probability–weighted odds ratio, 1.19; 95% CI, 1.06-1.33). There were no significant differences in ED visits among *CYP2D6* genotypic IMs or PMs vs NMs or UMs when testing all 4 drugs together. Among genotypic NMs, ED visits were more frequent among the individuals prescribed *CYP2D6* inhibitors (inverse probability–weighted odds ratio, 1.49; 95% CI, 1.32-1.68). In analyses by medication, drug interactions were important for all 4 medications, while genotype associations were significant only for hydrocodone, tramadol, and codeine.

**Conclusions and Relevance:**

In this cohort study, reduced *CYP2D6* activity was associated with increased ED visits among individuals treated with *CYP2D6*-metabolized opioids. This finding suggests that incorporating data on *CYP2D6* genotype and accounting for drug interactions in opioid prescribing may improve pain management and reduce ED visits.

## Key Points

**Question:**

Is the reduced function of cytochrome P450 2D6 (*CYP2D6*), via either *CYP2D6* genotype or *CYP2D6* inhibitor–mediated phenoconversion, associated with pain-related emergency department (ED) visits among patients taking *CYP2D6*-metabolized opioids?

**Findings:**

In this cohort study using a national dataset of 31 669 patients prescribed hydrocodone, tramadol, codeine, or oxycodone, patients who were phenotypic *CYP2D6* intermediate metabolizers or poor metabolizers, considering *CYP2D6* genotype and drug interactions, had significantly more pain-related ED visits than patients who were phenotypic ultrarapid metabolizers or phenotypic normal metabolizers.

**Meaning:**

This study suggests that reduced *CYP2D6* activity impairs the analgesic response of commonly used opioids metabolized via that pathway.

## Teaser

This cohort study evaluates whether individuals with reduced vs normal or high *CYP2D6* activity have more pain-related emergency department visits when pain taking CYP2D6-metabolized opioids.

## Introduction

In the US, approximately 100 million people experience chronic pain, with an annual cost between $560 billion and $635 billion, exceeding that of heart disease, cancer, or diabetes.^1^ Opioids metabolized by the enzyme cytochrome P450 2D6 (*CYP2D6*) include hydrocodone, tramadol, codeine, and oxycodone and represent most opioid prescriptions written in the US.^2^ *CYP2D6* bioactivates hydrocodone, tramadol, and codeine into active metabolites, and the parent compounds have little to no analgesic activity compared with the active metabolites.^3,4^ Both *CYP2D6* and *CYP3A4* metabolize oxycodone.^4,5^ As the parent drug oxycodone and some of the metabolites are active compounds, the data are equivocal on the association of *CYP2D6* with oxycodone’s analgesic response.^4,5^

*CYP2D6* is highly polymorphic, and more than 100 alleles have been identified.^6^ Variant alleles range from loss of function and no enzyme activity (with patients hereafter referred to as poor metabolizers [PMs]) to gene duplication and increased activity (with patients hereafter referred to as ultrarapid metabolizers [UMs]).^4,7^ Poor metabolizers experience a reduced analgesic response from *CYP2D6*-metabolized opioids compared with patients who are normal metabolizers (NMs) or intermediate metabolizers (IMs).^7^ In addition, there are commonly used drugs that the US Food and Drug Administration (FDA) has categorized as strong *CYP2D6* inhibitors (bupropion, fluoxetine, paroxetine, terbinafine, and quinidine) (eTable 1 in Supplement 1)^8^ that lead to the absence of *CYP2D6* function and moderate inhibitors (abiraterone, cinacalcet, mirabegron, duloxetine, lorcaserin, and rolapitant) that reduce *CYP2D6* activity by about half, with the potential to convert those with the NM phenotype to the PM phenotype or those with the UM phenotype to the IM phenotype.^4,9^ Many of the *CYP2D6* inhibitors are antidepressants, and data indicate that 15% to 25% of patients prescribed opioids are coprescribed a *CYP2D6* inhibitor.^10^

There is, however, little direct evidence of the association of *CYP2D6* genotype or inhibitors with analgesic activity of *CYP2D6*-metabolized opioids in terms of clinical outcomes. A recent clinical study found that patients prescribed a *CYP2D6*-metabolized opioid alongside a *CYP2D6*-inhibiting antidepressant had more pain-related emergency department (ED) visits, supporting the hypothesis that *CYP2D6* inhibition reduces the analgesic activity of opioids.^11^ No study from a clinical setting has included *CYP2D6* genetics and *CYP2D6* drug interactions because genetic data are typically not available within electronic health records (EHRs). In this study, we sought to evaluate the associations of *CYP2D6* genotype and *CYP2D6* inhibitor–mediated phenoconversion, alone and together, with the analgesic response of hydrocodone, tramadol, codeine, and oxycodone among patients with pain, as assessed by pain-related ED visits using the National Institutes of Health (NIH) All of Us Research Program (All of Us) data. We hypothesize that *CYP2D6* PMs and IMs, as defined by genotype, and those patients phenoconverted to these phenotypes by *CYP2D6* inhibitors will have worse pain control, as assessed by more frequent pain-related ED visits.

## Methods

### Data Source and Settings

We conducted a retrospective cohort study using EHR data and short-read whole-genome sequencing (SR-WGS) data from the All of Us Controlled Tier Dataset, version 7, using data from January 1, 2014, to December 31, 2022. This program, led by the NIH, creates one of the largest, most diverse nationwide biomedical data resources in the US.^12^ The All of Us institutional review board provides approval for all aspects of the All of Us program. All participants in All of Us provided written consent to participate in the research (eMethods in Supplement 1). The details of the All of Us research protocol have been previously published.^12^ This article complies with the Strengthening the Reporting of Observational Studies in Epidemiology (STROBE) reporting guideline for cohort studies.^13^

### Study Participants and Design

**Figure 1.**
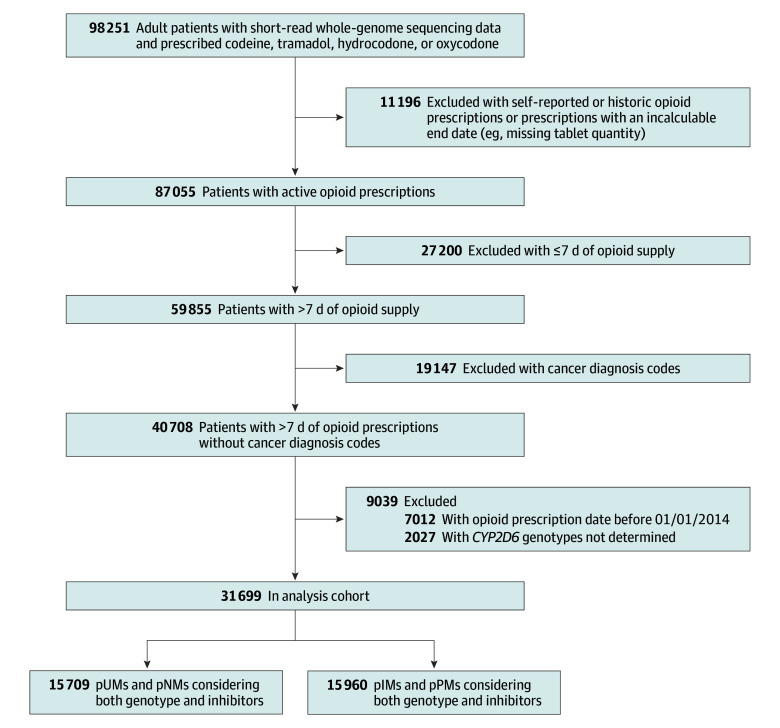
Flowchart of Study Cohort Identification Patients were referred to as phenotypic intermediate metabolizers (pUMs), phenotypic normal metabolizers (pNMs), phenotypic poor metabolizers (pPMs), and phenotypic ultrarapid metabolizers (pUMs) based on genotype and concomitant use of *CYP2D6* inhibitors.

**Figure 2.**
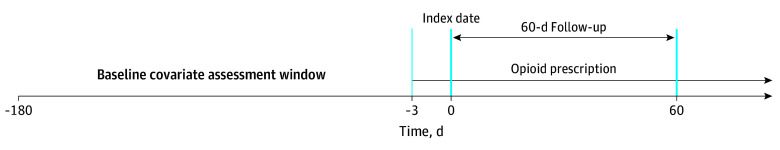
Graphical Representation of the Study Design The index date was set 3 days after the start date of the opioid prescription. Follow-up started from the index date and stopped at the end of 60 days or the end date of the opioid exposure, whichever occurred first. Baseline covariates were assessed for patients 180 days prior to the index date.

Inclusion criteria were adult patients (aged ≥18 years) who were prescribed at least 1 *CYP2D6*-metabolized opioid (hydrocodone, tramadol, codeine, or oxycodone) for at least 7 days from January 2014 to December 2022 and had all prescription-related and SR-WGS data available. Exclusion criteria were having cancer-related diagnosis codes in the 6 months prior to the index date and missing or indeterminate *CYP2D6* genotypes. Figure 1 outlines the measures used to define the study cohort. Index date (day zero) was 3 days after the date of the opioid prescription start date (Figure 2).

### *CYP2D6* Genotyping and Phenotype Assignment

To determine *CYP2D6* star alleles (haplotype patterns at the gene level that have been associated with protein activity levels) and diplotypes from the SR-WGS data, we used a consensus-based approach integrating results from multiple bioinformatic tools, including Aldy,^14^ Cyrius,^15^ PyPGx,^16^ and StellarPGx.^17^ Each of these tools uses distinct algorithms to address the complexities of *CYP2D6* genotyping. By combining the outputs of these tools, we generated more reliable *CYP2D6* genotype assignments. The consensus call used a majority agreement between haplotype callers while requiring a call from at least 2 tools. If a consensus call could not be made, an indeterminate genotype was reported. Each star allele was assigned an activity score, which was then used to define the inferred phenotype of the encoded protein based on genotype only or both genotype and *CYP2D6* inhibitors (Table 1) following the guidelines of Clinical Pharmacogenetics Implementation Consortium (CPIC).^4^

**Table 1.** *CYP2D6* Activity Score and Phenotype Adjusted After Consideration of *CYP2D6* Inhibitors

Allele and phenotypes	Activity score^a^
**Allele^b^**	
Functional (*1, *2 or *35)	1
Reduced functional (*9,*17,*29 or *41; *10)	0.5; 0.25
Nonfunctional (*3 through *8, *11 or *15)	0
**Phenotype based on genotype alone or based on genotype and inhibitors both** ^c^ ^,^ ^d^	
Ultrarapid metabolizers	>2.25
Normal metabolizers	≥1.25 to ≤2.25
Intermediate metabolizers	>0 to <1.25
Poor metabolizers	0

^a^ The activity score of *CYP2D6* is the sum of the values assigned to each allele.

^b^ Asterisks indicate star alleles of *CYP2D6*.

^c^ Phenotype based on genotype alone: phenotype is estimated based on the genotype-based activity score (sum of the values assigned to each allele).

^d^ Phenotype based on genotype and inhibitors both: here, phenotype is estimated based on the activity score considering the effect of drug interaction. So, *CYP2D6* activity score = inhibitor factor × genotype-based activity score; inhibitor factor = 0 for strong *CYP2D6* inhibitor, 0.5 for moderate *CYP2D6* inhibitor, and 1 for no *CYP2D6* inhibitor.

### Exposure

We assessed drug exposure based on the dates of prescriptions. In the All of Us EHR data, the medication directions (sig or instructions) were missing, so we used the most common directions of the prescriptions, as observed in a recent project (4 tablets a day),^11^ to calculate the end dates of the opioids. To accurately evaluate the exposure to concomitant opioid and *CYP2D6* inhibitor prescriptions, we implemented a stitching method in which we stitched or combined prescriptions of the same medication with a gap of fewer than 14 days between them and considered the end date of the last prescription as the end date of that opioid exposure. For antidepressant medications, we stitched or combined prescriptions of the same medication only if there was a gap of less than or equal to 3 days between them, and we considered the end date of the last prescription as the end date of that antidepressant medication prescription. Concomitant exposure was then determined based on the start date and calculated end date with a minimum of consecutive 3-day overlap. Patients who did not have any *CYP2D6* inhibitor prescription exposure in the last 180 days from the opioid start date and throughout the study period were considered not exposed to inhibitor prescriptions. We considered the first opioid prescriptions for patients who had multiple opioid prescriptions.

### Outcome and Follow-Up Periods

Our outcome variable for this study was an ED visit for pain. We identified the first pain-related ED visits based on pain-related diagnostic codes (eTable 2 in Supplement 1) that were captured in the ED during the follow-up period.^11^ Follow-up started from the index date and stopped at the end of 60 days or the end date of the opioid prescription, whichever occurred first (Figure 2).

### Covariates

We assessed 24 covariates (listed in Table 2) associated with patient demographic characteristics, comorbidities, comedications, and other factors that might have been associated with the outcome of ED visits for pain. Participants’ self-reported information about their race and ethnicity and sex from EHR data of All of Us was used to categorize them. These variables help control for confounding variables and understand demographics influences.

**Table 2.** Characteristics of the Patients

Characteristic	Patients, No. (%)^a^		*P* value
	pNM and pUM (n = 15 709)	pPM and pIM (n = 15 960)	
Age, mean (SD), y	50.6 (15.6)	51.8 (15.1)	<.001
Sex			
Female	10 227 (65.1)	10 838 (67.9)	<.001
Male	5482 (34.9)	5122 (32.1)	
Race and ethnicity			
Black	2968 (18.9)	2624 (16.4)	<.001
White	8747 (55.68)	10 570 (66.2)	
Other or none indicated^b^	3994 (25.4)	2766 (17.33)	
Pain diagnoses^c^^,^^d^			
Back pain	2031 (12.9)	2511 (15.7)	<.001
Pain in hand, leg, or joints	3991 (25.4)	4479 (28.1)	<.001
Rheumatoid arthritis	3547 (22.6)	4183 (26.2)	<.001
Headache (including migraine)	1416 (9.0)	1618 (10.1)	<.001
Neuropathic pain	1810 (11.5)	2330 (14.6)	<.001
Fibromyalgia	182 (1.2)	333 (2.1)	<.001
Injury	4033 (25.7)	4220 (26.4)	.12
Comorbidities^c^^,^^d^			
Depression	2036 (13.0)	3339 (20.9)	<.001
Psychoses	512 (3.3)	798 (5.0)	<.001
Anxiety	1775 (11.3)	2611 (16.4)	<.001
Opioid use disorder	168 (1.1)	216 (1.4)	.02
Diabetes	2424 (15.4)	2385 (14.9)	.23
Kidney failure	927 (5.9)	867 (5.4)	.07
Liver disease	846 (5.4)	821 (5.1)	.34
Medication history^c^^,^^d^			
Opioid or nonopioid pain medication	8581 (54.6)	8637 (54.1)	.37
Benzodiazepines and other sedative-hypnotics	4723 (30.1)	5430 (34.0)	<.001
Antipsychotics	2026 (12.9)	2829 (17.7)	<.001
CNS medications or stimulants	168 (1.1)	414 (2.6)	<.001
Skeletal muscle relaxants	3170 (20.2)	3613 (22.6)	<.001
Opioid-related medication use, MME/d	39.0 (89.6)	37.6 (83.1)	.13
Health care use (ED visits), mean (SD), No.^d^	0.32 (0.97)	0.31 (0.95)	.63

Abbreviations, CNS, central nervous system; ED, emergency department; MME, morphine milligram equivalent; pIM, phenotypic intermediate metabolizer; pNM, phenotypic normal metabolizer; pPM, phenotypic poor metabolizer; pUM, phenotypic ultrarapid metabolizer.

^a^ The patients were referred to as pUMs, pNMs, pIMs, and pPMs based on genetic activity and concomitant use of *CYP2D6* inhibitors.

^b^ Other includes Asian, Middle Eastern, Native Hawaiian, and mixed.

^c^ Patients could have more than 1 pain diagnosis, comorbidity, and medication history.

^d^ Within 6 months prior to the index date.

### Statistical Analysis

Statistical analysis was performed from July 1, 2023, to January 15, 2025. Demographic and baseline characteristics were compared between phenotypically UMs (pUMs) and phenotypically NMs (pNMs) vs phenotypically IMs (pIMs) and phenotypically PMss (pPM) as numbers and percentages (categorical variables) or mean (SD) values (continuous variables). Continuous variables were assessed for normality. Differences were assessed using χ^2^ tests or 2-sample *t* tests as appropriate.

We estimated propensity scores using logistic regression to model the probability of receiving the exposure of interest (pNMs or pUMs vs pIMs or pPMs) as a function of baseline patient characteristics. We used the propensity score to generate an inverse probability of treatment weight (IPTW) for each patient. The IPTW was calculated as the inverse of the propensity score for the exposed group and the inverse of 1 minus the propensity score for the comparison group. A candidate list of potential patient characteristics to be included in the propensity score models was determined a priori based on clinical knowledge and published literature of the characteristics likely to be associated with our outcome. Finally, 24 covariates associated with patient demographic characteristics, comorbidities, comedications, and other factors were included in the propensity score model. We assessed covariate balance before and after IPTW using the standardized mean difference, with a value of less than 0.1 considered balanced.^18^ After IPTW, the patient population was well balanced across all characteristics (eTable 3 in Supplement 1). For the primary analysis, logistic regression using IPTW was conducted to test the association between pNMs or pUMs and pIMs or pPMs with the presence of ED visit (yes or no) as a measure of pain control.

In addition to the primary analysis described, we evaluated the association of *CYP2D6* inhibitors with pain control among *CYP2D6* NMs as they do not have genetic impairments to the generation of the active metabolite (without confounding by genotype). Within this group, we tested ED visits among those with or without *CYP2D6* inhibitor prescriptions. Moreover, we evaluated the associations between *CYP2D6* phenotypes based on genotypes (genotypic NMs or UMs vs genotypic IMs or PMs) and ED visits among patients without any inhibitor prescription (not confounded by *CYP2D6* inhibitors).

We also conducted subgroup analyses for patients who were taking hydrocodone, tramadol, or codeine because these are drugs whose parent compounds mostly lack analgesic activity and are most likely to have an association with *CYP2D6* genotype or *CYP2D6* inhibitors. As the data are unclear on the association of *CYP2D6* with oxycodone response, we performed a separate subgroup analysis for the oxycodone cohort. For all the analyses, we used logistic regression to understand the association of *CYP2D6* genotype or inhibitor alone or together with pain-related ED visits. We reported unweighted odds ratios (ORs) and inverse probability (IP)–weighted ORs. Statistical significance was assessed using 95% CIs, where results were considered significant if the 95% CI did not include the null value. All *P* values were from 2-sided tests, and results were deemed statistically significant at *P* < .05. Data preprocessing was conducted in Python, version 3.10 (Python Software Foundation), and all analyses were performed using SAS, version 9.4 (SAS Institute Inc) within the All of Us Researcher Workbench. Some graphical presentations were prepared in Prism, version 10 (GraphPad Software).

## Results

### Study Population

The initial data pull yielded 98 251 adults who were prescribed at least 1 of the 4 opioids (hydrocodone, tramadol, codeine, or oxycodone); of these, 31 669 (mean [SD] age, 51.2 [15.4] years; 66.5% women and 33.5% men; 18.9% Black, 55.7% White, and 25.4% other race or ethnicity or none indicated) had active opioid prescriptions with a supply greater than 7 days and had available *CYP2D6* genotype data, representing the analysis cohort (Figure 1 and Table 2). Among them, 15.0% of patients were exposed to any of the FDA-defined *CYP2D6* strong or moderate inhibitors, while 85.0% were not. Basic demographic characteristics (eg, age, race and ethnicity, and sex) were complete with no missing data. We relied on EHR-recorded data, recognizing that certain factors—such as external prescriptions or diagnoses—may not be captured, limiting the assessment of missingness.

In the primary analysis, of 31 669 adults, 15 709 were pNMs or pUMs, and 15 960 were pPMs or pIMs. The demographic and clinical characteristics of the primary analysis are provided in Table 2. The distribution of various opioids and *CYP2D6* inhibitors among individuals is presented in eTable 4 in Supplement 1.

### Pain-Related ED Visits

#### Analysis of the Association of Both *CYP2D6* Inhibitors and Genotype With Pain-Related ED Visits

**Figure 3.**
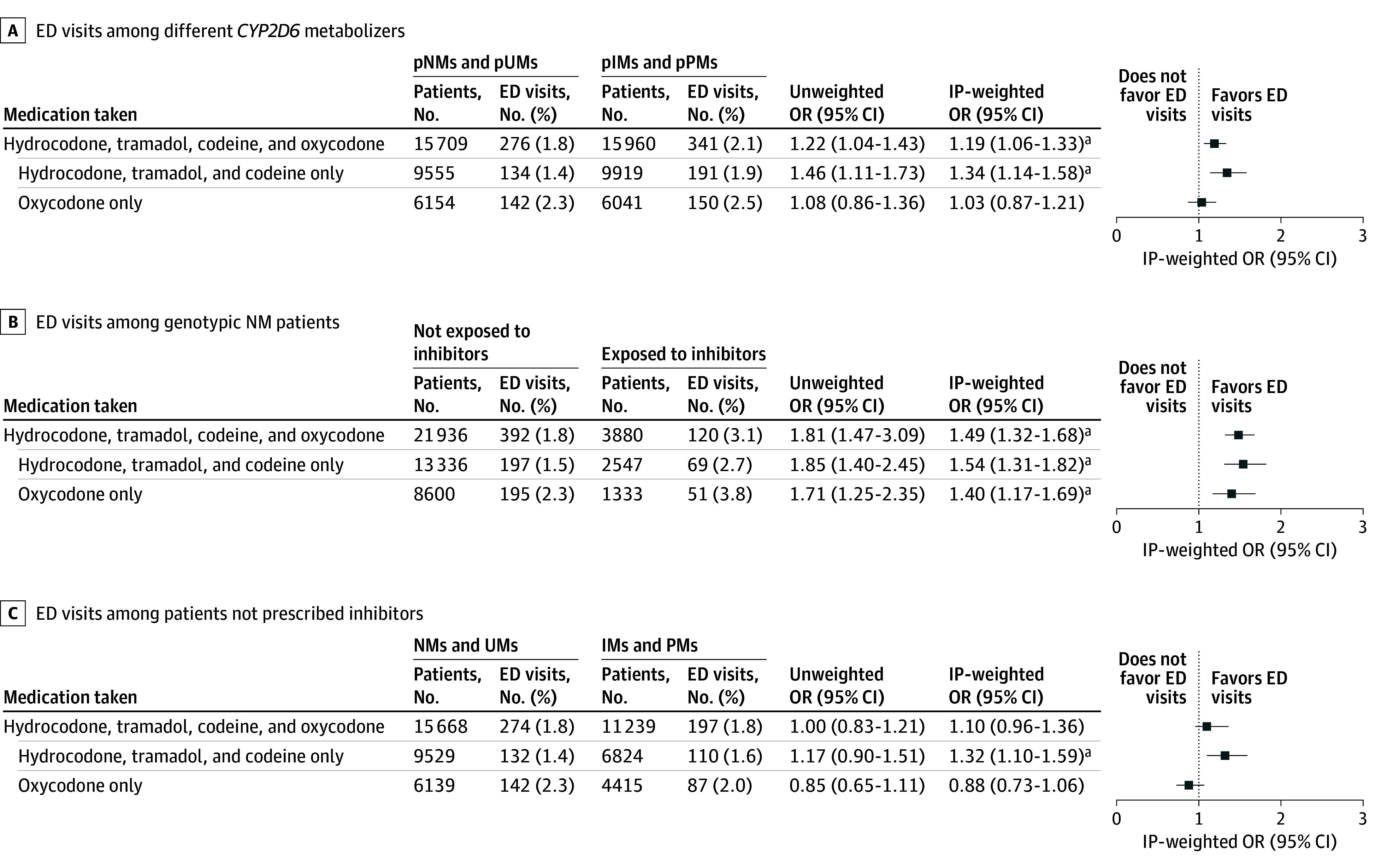
Association of *CYP2D6* Genotype and Inhibitors Alone and Together With Pain-Related Emergency Department (ED) Visits A, ED visits among different patients who were *CYP2D6* metabolizers: phenotypic normal metabolizers (pNMs) and phenotypic ultrarapid metabolizers (pUMs) vs phenotypic intermediate metabolizers (pIMs) and phenotypic poor metabolizers (pPMs), considering both *CYP2D6* inhibitors and genotype. B, ED visits among genotypic normal metabolizers (NMs) comparing those exposed to *CYP2D6* inhibitors vs those not exposed to *CYP2D6* inhibitors. C, Visits among patients not taking *CYP2D6* inhibitors comparing different *CYP2D6* metabolizers: genotypic NMs and ultrarapid metabolizers (UMs) vs genotypic intermediate metabolizers (IMs) and poor metabolizers (PMs). Patients were referred to as pUMs, pNMs, pPMs, and pIMs based on genotype and concomitant use of *CYP2D6* inhibitors; patients were referred to as UMs, NMs, IMs, and PMs based on genotype only. IP indicates inverse probability; OR, odds ratio. ^a^Statistically significant.

A higher percentage of patients who were pPMs or pIMs considering both *CYP2D6* genotype and inhibitors experienced pain-related ED visits compared with those who were pUMs and pPMs (2.1% vs 1.8%), with an IP-weighted OR of 1.19 (95% CI, 1.06-1.33) (Figure 3A). Among the different opioid subgroups, higher estimates were observed when examining the combination of hydrocodone, tramadol, and codeine only (IP-weighted OR, 1.34; 95% CI, 1.14-1.58). However, for oxycodone alone, there was no significant increase in ED visits between phenotypes (IP-weighted OR, 1.03; 95% CI, 0.87-1.21).

#### Analysis of the Association of *CYP2D6* Inhibitors Alone With Pain-Related ED Visits

To understand the association of *CYP2D6* inhibitors alone without confounding by genotype, we included only the NMs in this analysis. As shown in Figure 3B, this analysis yielded a significant association between ED visits and inhibitor prescription (IP-weighted OR, 1.49; 95% CI, 1.32-1.68). Subgroups based on different opioids yielded consistent findings.

#### Analysis of the Association of *CYP2D6* Genotype Alone With Pain-Related ED Visits

To examine the association of genotypes only, without the confounding influence of inhibitors, we included patients with no *CYP2D6* inhibitor prescriptions. Both genotypic IM or PM and UM or NM groups had identical pain-related ED visit rates (1.8%) (Figure 3C), and no significant association was observed (IP-weighted OR, 1.10; 95% CI, 0.96-1.36). In the hydrocodone, tramadol, and codeine subgroup, a significantly higher percentage of genotypic IMs and PMs experienced a pain-related ED visit compared with genotypic NMs and UMs (IP-weighted OR, 1.32; 95% CI, 1.10-1.59). Among oxycodone users, there were no differences in ED visits among genotype IMs and PMs compared with UMs and NMs.

## Discussion

Our primary analysis of 31 669 individuals taking hydrocodone, tramadol, codeine, or oxycodone showed that a significantly higher percentage of pIMs and pPMs, considering both *CYP2D6* genotype and inhibitors, experienced a pain-related ED visit compared with pUMs and pNMs. Among the combined subgroup of patients taking hydrocodone, tramadol, and codeine, we observed consistent results, but in the oxycodone-only analysis, we did not observe a significant increase in ED visits among pIMs or pPMs. This finding is consistent with existing literature that suggests *CYP2D6* may play a less critical role in the analgesic activity of oxycodone.

Previous studies indicate that the active metabolites biotransformed from hydrocodone, tramadol, and codeine by *CYP2D6* having higher affinity for the μ-opioid receptors are fully or largely responsible for the analgesic response those opioids provide.^3,19,20^ Our results suggest a clinically important association of reduced *CYP2D6* activity with patients who are phenotypically IMs or PMs while taking these drugs having increased pain-related ED visits.

Conversely, oxycodone is metabolized to oxymorphone by *CYP2D6* and noroxycodone by *CYP3A4*, which are further metabolized to noroxymorphone by the activity of *CYP2D6* and *CYP3A4*, respectively.^19,21^ Oxycodone, oxymorphone, and noroxymorphone are all active compounds; however, the exact association of the parent and different metabolites with the analgesic response is unknown.^22^ Data have generally suggested that *CYP2D6* activity may have smaller associations with the analgesic activity of oxycodone than that of other *CYP2D6*-metabolized opioids,^5,19^ and our results support that finding.

In 2 additional analyses, we aimed to separately examine the association of genotype and *CYP2D6* inhibitors alone with pain control, ensuring the absence of interference from the other. Among genotypic NMs, pain-related ED visits were more frequent among those prescribed *CYP2D6* inhibitors compared with patients not taking *CYP2D6* inhibitors, which is consistent with findings of a previous study^11^ and suggests that the coadministration of *CYP2D6* inhibitors with *CYP2D6*-metabolized opioids may impair opioid response, leading to increased pain-related ED visits. For patients not taking *CYP2D6* inhibitors, no overall association was observed across 4 opioids; however, hydrocodone, tramadol, or codeine users with IM and PM genotypes experienced more pain-related ED visits, whereas oxycodone users did not, indicating that oxycodone data diluted the overall analysis.

Unlike *CYP2D6* genotype effects, *CYP2D6* inhibitors might show a greater association with oxycodone response and pain-related ED visits by broadly disrupting drug metabolism and distribution. Although genetic variations are associated with *CYP2D6* activity—often offset by *CYP3A4*’s role—inhibitors block *CYP2D6* and may also inhibit *CYP3A4* (eg, by norfluoxetine, a main metabolite of fluoxetine), altering drug-drug interactions^23^ and/or changing transporter activities.^24^ These combined associations could lead to pronounced disruptions in oxycodone pharmacokinetics, amplifying their clinical association beyond that of genotype alone.

Given that ED visits are a major factor associated with health care costs, incorporating preemptive *CYP2D6* genotyping could proactively identify patients who might be at increased risk of poor pain control by the *CYP2D6*-metabolized opioids, improving pain management and reducing health care costs associated with ED visits. Clinical consideration of *CYP2D6* drug interactions could have a similar impact. Expanding clinical decision support tools to integrate both pharmacogenetics and drug-drug interactions would optimize pain management and potentially reduce opioid-related ED visits for pain.

Our data suggest that patients who are *CYP2D6* pIMs or pPMs and are prescribed hydrocodone, tramadol, or codeine have more ED visits than those with normal *CYP2D6* function. This finding implies they are experiencing inadequate pain control—which can be estimated based on the known pharmacokinetics of these drugs. These findings are consistent with current CPIC guidelines^4^ and add strength to the evidence suggesting that use of these data in the clinical setting may lead to improved patient outcomes. *CYP2D6* genotype data are readily available from multiple national references and other laboratories, and results are routinely reported as a phenotype (eg, IM). Use of pharmacogenetic data are optimized through pharmacogenetic clinical decision support tools, which are available in some EHR systems. Consideration of the drug interaction along with the genotype has not been optimized with tools in the EHR, but a calculator tool^25^ is available at PharmGKB^26^ that can calculate the *CYP2D6* phenotype based on genotype and drug interactions. Although genotype is immutable, the inhibitor drug prescriptions can change over time, so genotype must be reassessed with each new opioid prescription.^4,11,27^ Although our data with oxycodone did not reveal an association with genotype alone, when considering *CYP2D6* inhibitors, we did observe increased ED visits. Thus, consideration of drug interactions, and perhaps genotype, may also be reasonable with oxycodone.

### Strengths and Limitations

This study has several strengths. First, to our knowledge, this is the first population-based clinical cohort study investigating the association of reduced *CYP2D6* activity, considering *CYP2D6* genotypes and inhibitor alone (isolating the associations of inhibitors and genetics) and together, with pain control by the *CYP2D6*-metabolized opioids. Second, use of data from All of Us allowed for a diverse, nationwide sample, enhancing generalizability. Third, we included a large cohort with *CYP2D6* genotype data obtained from SR-WGS data available in All of Us.

This study also has several limitations. We used EHR data from All of Us, which may include misclassifications and incomplete records from various health care systems. Some participants may have received care outside the shared EHR network, limiting the assessment of missing data. Emergency department visits may be underreported, as people are likely to go to an ED close to where they live. However, this would be expected to bias toward the null. Prescription directions were unavailable, requiring assumptions based on prior studies.^11^ Again, actual consumption of the medications could not be verified. Differences between comparison groups may arise from combining individuals with different pain problems,^11^ but propensity scoring ensured balance, and all ORs were IP weighted.

## Conclusions

In this cohort study, reduced *CYP2D6* activity was observed to be associated with increased pain-related ED visits among those treated *CYP2D6*-metabolized opioids. Incorporating *CYP2D6* genotyping and accounting for drug interactions in opioid prescribing may improve pain management and reduce ED visits.

## Notes

Supplement 1. **eMethods.** Patient Consent and Data From All of Us Research Program **eTable 1**. List of CYP2D6 Strong and Moderate Inhibitors **eTable 2.** List of Emergency Department (ED) Visit SNOMED Standard Concept Name and Codes **eTable 3**. Characteristics of the Patients Before and After Inverse Probability of Treatment Weighted (IPTW) Balancing **eTable 4.** Distribution of the Different Medications in the Study Population

Supplement 2.
Data Sharing Statement
