# Supplementary material for: CYP2D6 Phenotypes and Emergency Department Visits Among Patients Receiving Opioid Treatment
Source: JAMA Netw Open. 2025 Jul 28;8(7):e2523543. doi: 10.1001/jamanetworkopen.2025.23543 (PMC12305384; doi:10.1001/jamanetworkopen.2025.23543)
Supplement: Supplement 1. — eMethods. Patient Consent and Data From All of Us Research Program eTable 1. List of CYP2D6 Strong and Moderate Inhibitors eTable 2. List of Emergency Department (ED) Visit SNOMED Standard Concept Name and Codes eTable 3. Characteristics of the Patients Before and After Inverse Probability of Treatment Weighted (IPTW) Balancing eTable 4. Distribution of the Different Medications in the Study Population [file jamanetwopen-e2523543-s001.pdf]

Supplementary Online Content

Nahid NA, McDonough CW, Wei YJJ, et al. *CYP2D6* phenotypes and emergency department visits among patients receiving opioid treatment. *JAMA Netw Open.* 2025;8(7):e2523543. doi:10.1001/jamanetworkopen.2025.23453

eMethods. Patient Consent and Data From All of Us Research Program

eTable 1. List of CYP2D6 Strong and Moderate Inhibitors

eTable 2. List of Emergency Department (ED) Visit SNOMED Standard Concept Name and Codes

eTable 3. Characteristics of the Patients Before and After Inverse Probability of Treatment Weighted (IPTW) Balancing

eTable 4. Distribution of the Different Medications in the Study Population

This supplementary material has been provided by the authors to give readers additional information about their work.

eMethods. Patient Consent and Data From All of Us Research Program

"*The Registered Tier and Controlled Tier data available on the Research Hub contains data from participants who have consented to be involved in the* All of Us *Research Program, including data from electronic health records (EHRs), surveys, and physical measurements. All data available to researchers has had direct identifiers removed and has been further modified to minimize re-identification risks. This includes removing all explicit identifiers in both EHRs and participant provided information, all free-text fields, geolocation data smaller than U.S. state level, living situations, race and ethnicity subcategories, active duty military status, cause of death, and diagnosis codes subject to public knowledge. Additionally, the select demographic fields are generalized. Also, all dates are systematically shifted backwards by a random number between 1 and 365, and data from participants over the age of 89 are removed. The* All of Us *Research Program data will be accessed for research strictly using the Researcher Workbench (researchallofus.org). External data can be brought into this secure environment; however, researchers are restricted from importing any individually identifiable information and from row-level linkage of the external data. Data searches, cohort building, and analysis will solely take place on the Researcher Workbench, a secure cloud-based resource with statistical analysis software available for use with* All of Us *data. Researchers are granted access to the Researcher Workbench after their affiliated institution signs a Data Use and Registration Agreement, and they create an account, including setting up two-factor authentication, verify their identity through Login.gov or ID.me, complete the* All of Us *Responsible Conduct of Research training, and sign a Data User Code of Conduct, which prohibits any re-identification of* All of Us *participants. For more information, please visit researchallofus.org.*"

Source: https://www.researchallofus.org/faq/do-i-need-institutional-review-board-irb-approval-from-my-own-institution-in-order-to-access-this-data-through-the-researcher-workbench/

© 2025 Nahid NA et al. *JAMA Network Open.*

eTable 1. List of CYP2D6 Strong and Moderate Inhibitors

**CYP2D6 inhibitors**	**Strong inhibitors**	Bupropion, fluoxetine, paroxetine, terbinafine, quinidine
	**Moderate inhibitors**	Abiraterone, cinacalcet, mirabegron, duloxetine, lorcaserin, rolapitant

fda.gov. U.S. Food and Drug Administration. Drug Development and Drug Interactions: Table of Substrates, Inhibitors and Inducers. https://www.fda.gov/drugs/developmentapprovalprocess/developmentresources/druginteractionslabeling/ucm093664.htm Accessed on 08/06/2022.

© 2025 Nahid NA et al. *JAMA Network Open.*

eTable 2. List of Emergency Department (ED) Visit SNOMED Standard Concept Name and Codes

Standard concept name	Standard concept code
Headache	25064002
Chronic pain	82423001
Low back pain	279039007
Osteoarthritis	396275006
Backache	161891005
Neck pain	81680005
Shoulder joint pain	267949000
Pain in limb	90834002
Hip pain	49218002
Right lower quadrant pain	301754002
Right upper quadrant pain	301717006
Left lower quadrant pain	301716002
Pain in left lower limb	287047008
Arthralgia of the ankle and/or foot	267954009
Pain in right lower limb	287048003
Pain in right knee	316931000119104
Fibromyalgia	203082005
Pain in left knee	316791000119102
Rheumatoid arthritis	69896004
Knee pain	30989003
Pain in pelvis	30473006
Osteoarthritis of knee	239873007
Muscle pain	68962001
Wrist joint pain	202482009
Sciatica	23056005
Pain in thoracic spine	267981009
Pain	22253000
Knee joint effusion	202381003
Lumbago with sciatica	202794004
Pain in left foot	316751000119107
Pain in right foot	316891000119107
Left upper quadrant pain	301715003
Pleuritic pain	2237002
Cervical spondylosis without myelopathy	267970006
Pain in left arm	287045000
Persistent pain following procedure	279047007
Lumbar spondylosis	239880009
Generalized aches and pains	82991003
Elbow joint pain	202480001

© 2025 Nahid NA et al. *JAMA Network Open.*

Joint pain of pelvic region	713413001
Pain in right arm	287046004
Osteoarthritis of hip	239872002
Pain in lower limb	10601006
Chronic pain syndrome	373621006
Tension-type headache	398057008
Acute pain	274663001
Joint pain	57676002
Pain in eye	41652007
Pain in right hand	316911000119109
Localized osteoarthrosis uncertain if primary ...	90860001
Jaw pain	274667000
Hand pain	53057004
Renal colic	7093002
Periumbilical pain	443503005
Localized, primary osteoarthritis of the shoul...	201831003
Pain in finger	18876004
Thigh pain	78514002
Localized, primary osteoarthritis of the ankle...	201837004
Degeneration of lumbar intervertebral disc	26538006
Pain in throat	162397003
Pain due to neoplastic disease	98921000119102
Pain in toe	285365001
Pain of breast	53430007
Lumbosacral spondylosis without myelopathy	48210000
Trigeminal neuralgia	31681005
Degeneration of cervical intervertebral disc	69195002
Arthropathy of knee joint	428724006
Pain provoked by trauma	707809009
Degeneration of intervertebral disc	77547008
Acute postoperative pain	107401000119105
Migraine with aura	4473006
Localized, primary osteoarthritis of the hand	201834006
Pain in forearm	444899003
Thoracic spondylosis without myelopathy	267971005
Ankle joint effusion	202383000
Headache disorder	230461009
Hand joint pain	202472008
Suppurative arthritis	372939007
Polymyalgia rheumatica	65323003
Neuralgia	16269008
Status migrainosus	230467008

© 2025 Nahid NA et al. *JAMA Network Open.*

Inflammatory polyarthropathy	417373000
Foot pain	47933007
Psoriasis with arthropathy	33339001
Pain due to internal prosthetic device	213134006
Spondylosis without myelopathy	68859000
Shoulder pain	45326000
Posttraumatic headache	54012000
Idiopathic osteoarthritis	239862000
Spondylosis	8847002
Ankylosing spondylitis	9631008
Pain in upper limb	102556003
Localized, primary osteoarthritis of the wrist	313257005
Synovitis and tenosynovitis	202900007
Elbow joint effusion	202373004
Arthropathy of pelvis	128268005
Refractory migraine	423894005
Acquired hallux valgus	65358001
Bilateral earache	162359003
Arthropathy of right knee joint	774130003
Arthropathy of left knee joint	774129008
Pain in scrotum	20502007
Traumatic dislocation of hip joint	125621009
Joint effusion of ankle AND/OR foot	4819006
Pain in testicle	63901009
Degeneration of lumbosacral intervertebral disc	60937000
Acute posttraumatic headache	122751000119105
Complex regional pain syndrome	128200000
Arthralgia of the pelvic region and thigh	267952008
Arthritis of shoulder region joint	443798008
Arthritis of spine	371082009
Disorder of joint of ankle and/or foot	442246002
Cervico-occipital neuralgia	71760005
Pain disorder with psychological factor	381000119107
Lumbar spondylosis with myelopathy	67437007
Osteoarthrosis of the carpometacarpal joint of...	37895003
Multiple joint pain	35678005
Dislocation of hip joint prosthesis	314201008
Thoracic back pain	279038004
Gouty arthropathy	190828008
Neuropathic arthropathy due to type 2 diabetes...	781000119106
Greater trochanteric pain syndrome	7674000
Seropositive rheumatoid arthritis	239791005

© 2025 Nahid NA et al. *JAMA Network Open.*

Derangement of knee	63643000
Pain of right testicle	16675201000119100
Arthralgia of temporomandibular joint	91943004
Bunion	415692008
Pain of left testicle	16675301000119100
Degenerative joint disease of pelvis	445478004
Localized, primary osteoarthritis of elbow	313259008
Closed traumatic dislocation of joint of shoul...	22911007
Wrist joint effusion	202375006
Inflammation of sacroiliac joint	55146009
Lateral epicondylitis	202855006
Atypical facial pain	71303008
Hemarthrosis of knee	202413005
Pain of intercostal space	735940001
Arthropathy of joint of hand	428360003
Prepatellar bursitis	17059001
Discitis	2304001
Pain in spine	48926009
Pain in right hip joint	316921000119102
Migraine variants	193030005
Arthritis	3723001
Pain in wrist	56608008
Osteoarthrosis involving multiple sites but no...	33262002
Degeneration of thoracic intervertebral disc	68675004
Chondrocalcinosis of knee joint	442942009
Chronic interstitial cystitis	197834003
Degenerative joint disease involving multiple ...	201819000
Seronegative rheumatoid arthritis	239792003
Phantom limb syndrome with pain	5771000119106
Rest pain	52598005
Cervical spondylosis	387800004
Refractory migraine without aura	423279000
Bacterial arthritis	48245008
Degenerative joint disease of shoulder region	67315001
Arthropathy of the ankle AND/OR foot associate...	430428000
Acute low back pain	278862001
Flank pain	247355005
Hammer toe	122481008
Chronic tension-type headache	230471006
Pain of left lower leg	316801000119101
Juvenile rheumatoid arthritis	410795001
Traumatic dislocation of interphalangeal joint...	446091003

© 2025 Nahid NA et al. *JAMA Network Open.*

Traumatic amputation, finger, through metacarp...	210625001
Cluster headache	193031009
Foot joint effusion	298154000
Neurogenic claudication	303081002
Episodic tension-type headache	230470007
Hemiplegic migraine	59292006
Complex regional pain syndrome of lower limb	734986006
Glossodynia	30731004
Effusion of joint of hand	16711001
Localized, primary osteoarthritis	201829007
Charcot's arthropathy	359554008
Arthralgia of the upper arm	267950000
Thoracic spondylosis with myelopathy	10948005
Arthritis of knee	371081002
Synovitis/tenosynovitis - hand	288213002
Headache caused by drug	294091000119104
Toothache	27355003
Pain of right lower leg	316941000119108
Disorder of hip joint	428097001
Knee pyogenic arthritis	239777004
Closed traumatic dislocation acromioclavicular...	208759001
Pelvic and perineal pain	274671002
Dislocation of metacarpophalangeal joint	312844001
Degenerative joint disease of ankle AND/OR foot	82300000
Current tear of semilunar cartilage	19494006
Mallet finger	64298006
Bursitis of knee	111243002
Dislocation of patellofemoral joint	263029007
Hip joint effusion	202379000
Patellar tendonitis	37785001
Traumatic dislocation of joint of finger	125619004
Pain of right wrist	316991000119100
Refractory migraine with aura	423683008
Disorder of wrist joint	428107009
Pain in elbow	74323005
Complex regional pain syndrome of upper limb	712537009
Lumbar discitis	202752002
Chronic polyarticular juvenile rheumatoid arth...	1961000
Thoracic spondylosis	387802007
Meralgia paresthetica	85007004
Pain finding at anatomical site	279001004
Osteoarthritis of right knee joint	323321000119100

© 2025 Nahid NA et al. *JAMA Network Open.*

Pain of left wrist	316851000119102
Osteoarthritis of left knee joint	323301000119109
Closed traumatic dislocation of joint of finger	75137002
Medial epicondylitis	53286005
Degeneration of thoracolumbar intervertebral disc	91240008
Staphylococcal arthritis	111820003
New daily persistent headache	121021000119105
Paroxysmal hemicrania	443094001
Chronic intractable migraine without aura	124171000119105
Tear of lateral meniscus of knee	302933001
Vascular headache	128187005
Migraine without aura, not refractory	425007008
Chronic low back pain	278860009
Degenerative joint disease of hand	22193007
Primary thunderclap headache	122731000119104
Pain of right eye	333601000119102
Acute headache	735938006
Closed traumatic dislocation of interphalangea...	125802004
Intermittent claudication of left lower limb c...	12237071000119100
Rheumatoid lung disease with rheumatoid arthritis	319841000119107
Effusion of joint of left knee	306891000119105
Traumatic dislocation of joint of wrist	125618007
Pain from metastases	315241008
Traumatic dislocation of ankle joint	125622002
Chronic postoperative pain	109771000119103
Reactive arthritis triad	67224007
Closed traumatic dislocation of hip	208892001
Intermittent claudication due to atheroscleros...	792845002
Current tear of medial cartilage AND/OR menisc...	307945003
Intermittent claudication of bilateral lower l...	12236951000119100
Infective arthritis of shoulder region	428437005
Adhesive capsulitis of right shoulder	301971000119109
Chondrocalcinosis of wrist joint	442925003
Traumatic dislocation of elbow joint	125617002
Pain of left hand	316771000119103
Intermittent claudication	63491006
Ankle pain	247373008
Episodic cluster headache	230472004
Pain of truncal structure	301366005
Lumbosacral spondylosis with radiculopathy	202693003
Migraine with persistent visual aura	699314009
Closed traumatic dislocation of patellofemoral...	208929003

© 2025 Nahid NA et al. *JAMA Network Open.*

Chronic post-traumatic headache	230477005
Pain in calf	300954003
Thoracic discitis	202748002
Post-herpetic trigeminal neuralgia	17974002
Closed traumatic dislocation of elbow joint	2651006
Left flank pain	162049009
Adhesive capsulitis of left shoulder	301981000119107
Subluxation of patellofemoral joint	263059002
Arthropathy of elbow	429554009
Disorder of ligament of right ankle joint	306051000119107
Traumatic dislocation of joint of foot	125623007
Pain of left thigh	316821000119105
Pain of left eye	339211000119104
Monoarthritis of knee	698474005
Pain at rest of right lower limb co-occurrent ...	12237231000119100
Acute back pain with sciatica	247366003
Tear of meniscus of knee	239720000
Primary localized osteoarthrosis of pelvic region	77994009
Derangement of medial meniscus	111222003
Intermittent claudication of right lower limb ...	12237191000119100
Pain due to varicose veins of lower extremity	706883004
Lumbago co-occurrent with right-side sciatica	313501000119105
Closed traumatic dislocation of joint of foot	63141004
Effusion of right knee joint	306971000119104
Osteoarthritis of wrist	239867006
Bladder pain	15803009
Central pain syndrome	426566004
Closed traumatic dislocation of knee joint	35106007
Traumatic closed dislocation of temporomandibu...	8135006
Intractable headache following trauma	290431000119101
Ophthalmoplegic migraine	49605003
Loose body in knee	81512004
Acquired hallux malleus	45636002
Arthritis of hand	448589005
Arthritis of hip	68449006
Dislocation of carpometacarpal joint	281503004
Pain of right thigh	316961000119107
Closed traumatic dislocation ankle joint	208981003
Chondromalacia of patella	36071006
Infective arthritis	396234004
Rheumatoid arthritis - hand joint	287007001
Rheumatoid arthritis of multiple joints	287006005

© 2025 Nahid NA et al. *JAMA Network Open.*

Biliary colic	37389005
Idiopathic stabbing headache	230481005
Hyperesthesia	14151009
Tibial collateral ligament bursitis	44245003
Pyogenic arthritis of the ankle and/or foot	267882003
Dislocation of toe joint	263030002
Numbness	44077006
Acute thoracic back pain	279035001
Pain at rest of left lower limb co-occurrent a...	12237111000119100
Infective arthritis of ankle and/or foot	444999002
Relapsing polychondritis	72275000
Pain in bilateral legs	15634511000119100
Vaginal pain	38343000
Closed traumatic dislocation of joint of wrist	38556006
Hemarthrosis of elbow	202405009
Numbness and tingling sensation of skin	101000119102
Rebound tenderness of right iliac fossa	301419005
Enteropathic arthritis	9350004
Post traumatic osteoarthritis	699262001
Chondromalacia	63198006
Postoperative pain	213299007
Acquired hallux rigidus	6654000
Primary chronic gout without tophus of ankle a...	309661000119108
Chondrocalcinosis of joint of ankle AND/OR foot	442941002
Thoracic nerve root pain	103015000
Osteoarthritis of multiple joints	268054009
Erosive osteoarthrosis	201826000
Pyogenic arthritis of shoulder region	36678001
Derangement of meniscus	111224002
Cervical discitis	202744000
Infective arthritis of right knee	1073511000119100
Right flank pain	162050009
Chronic back pain	134407002
Effusion of joint of pelvic region	9363005
Streptococcal arthritis	51646002
Traumatic dislocation of knee joint	58320001
Otalgia of right ear	1091931000119100
Acute ankle pain	51741000119105
Subluxation of wrist joint	263053001
Lumbosacral spondylosis	123798002
Closed posterior dislocation of elbow	4273008
Osteoarthritis of right hip joint	323311000119107

© 2025 Nahid NA et al. *JAMA Network Open.*

Juvenile seronegative polyarthritis	410797009
Post-herpetic polyneuropathy	76462000
Pelvic congestion syndrome	39402007
Pain of right upper arm	316981000119103
Infective arthritis of wrist	445525001
Localized, secondary osteoarthritis of the sho...	201849003
Pain around eye	86260003
Pain in finger of right hand	316881000119109
Hemicrania continua	443095000
Pain of toe of right foot	316971000119101
Hemarthrosis of right knee	299161000119109
Metatarsalgia	10085004
Rheumatoid arthritis - ankle and/or foot	287008006
Pain in finger of left hand	316741000119105
Pain of left upper arm	316841000119104
Traumatic dislocation of joint of thumb	125620005
Osteoarthritis of bilateral hip joints	112991000119105
Primary coxarthrosis, bilateral	201839001
Traumatic arthropathy-knee	201952005
Traumatic arthropathy of the ankle and/or foot	201938008
Radicular pain	11679003
Osteoarthritis of joint of right shoulder region	318721000119100
Rectal pain	77880009
Complex regional pain syndrome, type II, lower...	408749000
Traumatic amputation, thumb, through metacarpo...	210614005
Rib pain	297217002
Sore throat symptom	267102003
Rheumatoid arthritis of knee	201777003
Osteoarthritis of left hip joint	323291000119108
Acquired deformity of hip	67321002
Osteoarthritis of joint of left shoulder region	318671000119108
Pain of left calf	1076771000119100
Psoriatic arthritis mutilans	10629311000119100
Infection of intervertebral disc - pyogenic	202756004
Disorder of ligament of left ankle joint	305981000119109
Paresthesia of upper limb	95673003
Subluxation of ankle joint	263060007
Thoracic spondylosis with radiculopathy	202688001
Boutonnière deformity	43234007
Pain of right forearm	774133001
Dislocation of hip joint	157265008
Crystal arthropathy	18834007

© 2025 Nahid NA et al. *JAMA Network Open.*

Localized osteoarthrosis	33952002
Disorder of patellofemoral joint	239732001
Acquired genu varum	64925008
Giant cell arteritis with polymyalgia rheumatica	239938009
Closed anterior dislocation of elbow	3019000
Hypesthesia	397974008
Rebound tenderness of epigastrium	301412001
Osteoarthritis of elbow	239866002
Pain of right calf	1076761000119100
Wrist pyogenic arthritis	239778009
Arthritis of wrist	1771000119109
Synovitis/tenosynovitis - shoulder	287017006
Chronic neck pain	1121000119107
Dislocation of elbow joint	417558002
Arthritis of left knee caused by bacteria	1073331000119100
Inguinal pain	102570003
Subluxation of knee joint	263058005
Ankylosis of lumbosacral joint	87823004
Gout of hand due to renal impairment	308801000119100
Crystal arthropathy of knee	429420005
Cough headache syndrome	95658004
Acquired genu valgum	52012001
Vesical tenesmus	784285002
Osteoarthritis of joint of right ankle and/or ...	318691000119109
Pain in face	95668009
Monoarthritis	699462004
Orthostatic headache	445511002
Pain at rest due to peripheral vascular disease	428171009
Headache associated with sexual activity	103010005
Elbow pyogenic arthritis	239779001
Subluxation of toe joint	263062004
Rheumatoid arthritis of elbow	201769002
Sarcoid arthropathy	361197009
Localized, secondary osteoarthritis	201847001
Intrapelvic protrusion of acetabulum	59606006
Derangement of lateral meniscus	21333004
Pain of bilateral eyes	344961000119109
Open dislocation of finger	54420005
Paresthesia of lower extremity	429783005
Chondrocalcinosis of joint of hand	442884002
Trigeminal autonomic cephalalgia	449814007
Vulvodynia	238968009

© 2025 Nahid NA et al. *JAMA Network Open.*

Chronic cluster headache	230473009
Pain of left forearm	316761000119109
Traumatic dislocation of joint of cervical ver...	44264009
Traumatic dislocation of interphalangeal joint...	733256000
Pain in female pelvis	426702003
Acute gout	770924008
Chondrocalcinosis of elbow joint	443001001
Recurrent dislocation of hip	429190007
Rheumatism	396332003
Subluxation of radial head	417109008
Rheumatoid arthritis of wrist	201771002
Subluxation of hip joint	263057000
Bilateral atherosclerosis of lower limbs with ...	15649941000119100
Pain of toe of left foot	316831000119108
Episodic paroxysmal hemicrania	294031000119103
Closed traumatic dislocation of metacarpophala...	29818001
Localized, secondary osteoarthritis of the ank...	201855008
Degeneration of cervicothoracic intervertebral...	43132002
Heberden node	371598009
Effusion of joint of left ankle	306841000119102
Congenital deformity of hip joint	2749000
Congenital deformity of knee joint	61293001
Menstrual migraine	23186000
Dislocation of digit of hand	827108008
Migraine variants, not intractable	424699007
Left inguinal pain	15629941000119100
Hematuria co-occurrent and due to chronic inte...	367661000119102
Bacterial arthritis of hip	431606003
Osteoarthritis of joint of right hand	318711000119107
Bilateral lower limb atherosclerosis pain at r...	12236991000119100
Articular cartilage disorder of hip	428861009
Osteoarthritis of joint of left hand	318661000119102
Arthropathy of the hip associated with a neuro...	432463002
Closed anterior dislocation of proximal end of...	41359009
Closed traumatic dislocation of interphalangea...	21949009
Painful mouth	102616008
Ankylosis of joint of foot	429301007
Gout of wrist due to renal impairment	298961000119102
Hemarthrosis of left knee	299081000119101
Hemarthrosis of hip	202411007
Effusion of joint of right ankle	306921000119100
Monoarthritis of ankle and/or foot	698479000

© 2025 Nahid NA et al. *JAMA Network Open.*

Otogenic otalgia	74123003
Closed traumatic dislocation of tarsometatarsa...	41061006
Pain in both feet	15634431000119100
Tight chest	23924001
Ureteric colic	17329003
Neuropathic pain	247398009
Knee joint ankylosis	202317007
Flexion deformity of finger	203554005
Crystal arthropathy of ankle AND/OR foot	75468006
Acquired hallux varus	16123003
Rheumatoid arthritis of hip	201775006
Chronic gouty arthritis	68451005
Chronic headache disorder	431237007
Open dislocation of interphalangeal joint of hand	19241004
Rheumatoid arthritis of shoulder	201766009
Primary chronic gout without tophus of knee	309741000119104
Crystal arthropathy of multiple sites	20075001
Numbness of face	309557009
Post-thoracotomy pain syndrome	239175003
Pyogenic arthritis of pelvic region	19375000
Gouty arthritis of the ankle and/or foot	201669005
Pain in buttock	279043006
Pyogenic arthritis of hand	61112000
Cervicogenic headache	279016001
Referred otalgia	12336008
Pes anserinus tendinitis and bursitis	202868003
Intractable headache caused by drug	294081000119102
Pain of left hip joint	316781000119100
Paresthesia of left upper limb	15973661000119100
Chronic pain of right upper limb	15743521000119100
Acute postthoracotomy pain syndrome	444227004
Traumatic amputation, thumb, through interphal...	210616007
Synovitis/tenosynovitis - multiple joints	287016002
Osteoarthritis of joint of left ankle and/or foot	737057000
Monoarthritis of elbow	698478008
Acquired coxa valga	16979000
Systemic onset juvenile chronic arthritis	201796004
Gout of knee due to renal impairment	308821000119109
Osteoarthritis of hip due to dysplasia	712504007
Musculoskeletal pain	279069000
Vertebrogenic pain syndrome	398997008
Pain of nose	225564006

© 2025 Nahid NA et al. *JAMA Network Open.*

Spinal and epidural anesthesia-induced headach...	200073003
Spinal and epidural anesthesia-induced headach...	200076006
Solitary sacroiliitis	239815007
Inadequate pain control	704675005
Sinus headache	4969004
Derangement of posterior horn of medial meniscus	5313005
Calcium pyrophosphate deposition disease	239832006
Closed traumatic dislocation of distal radioul...	57467003
Intermittent pain	314642004
Transformed migraine	427419006
Anorectal pain	197232005
Paresthesia of left lower limb	15634841000119100
Traumatic dislocation of pelvis	125613003
Chondrocalcinosis of hip joint	443002008
Numbness of lower limb	309537005
Old anterior cruciate ligament disruption	202110002
Rebound tenderness	35611005
Chondrocalcinosis of shoulder region	442928001
Left trigeminal neuralgia	12242071000119100
Traumatic dislocation of joint of lumbar vertebra	129166009
Frontal headache	267096005
Photophobia	409668002
Elbow pathological dislocation	239761000
Traumatic arthropathy	58188004
Derangement of anterior horn of medial meniscus	9771006
Gonococcal infection of joint	44743006
Diffuse cervicobrachial syndrome	33420007
Closed traumatic dislocation of tarsal joint	55126004
Otalgia of left ear	1089321000119100
Synovitis/tenosynovitis - knee	287021004
Effusion of joint of left elbow	306851000119100
Monoarthritis of hand	698475006
Gout of hand caused by drug	306631000119107
Malignant bone pain	282743009
Hemarthrosis of left elbow	299041000119106
Closed posterior dislocation of hip	63975004
Tendonitis of left patellar tendon	317511000119102
Monoarthritis of wrist	698476007
Complex regional pain syndrome type I	734947007
Closed dislocation of sacrum	269129003
Prostatic pain	36729000
Closed traumatic dislocation of joint	445852002

© 2025 Nahid NA et al. *JAMA Network Open.*

Articular gout	48440001
Complicated migraine	193039006
Gout of elbow due to renal impairment	308791000119101
Pain in thumb	300955002
Effusion of joint of left hip	306881000119107
Subluxation of thumb	263055008
Fistula of elbow joint	426407008
Joint pain in left hand	1076731000119100
Acute polyarticular juvenile rheumatoid arthritis	75822003
Choking sensation	373909009
Traumatic rupture of symphysis pubis	208207008
Recurrent dislocation of the patellofemoral joint	202246002
Recurrent dislocation of knee	202245003
Epidemic pleurodynia	83264000
Recurrent dislocation of ankle AND/OR foot	50952009
Bacterial arthritis of elbow	431263002
Dislocation of hand joint	314664008
Psoriatic arthritis with spine involvement	200956002
Gouty tophus of hand	190842000
Disorder of ankle joint	428776005
Ureteric pain	274280006
Subluxation of finger	263054007
Pyogenic arthritis of hip	372941008
Swan-neck deformity	23060008
Suprapubic pain	162053006
Swallowing painful	30233002
Cervical nerve root pain	103014001
Generalized osteoarthritis of the hand	267889007
Infective arthritis of joint of hand	428385007
Short-lasting unilateral neuralgiform headache...	725058003
Bursitis of right knee	1076181000119100
Secondary localized osteoarthrosis of pelvic r...	25343008
Derangement of posterior horn of lateral meniscus	77860008
Sacroiliac joint pain	202487003
Hemarthrosis of the ankle and/or foot	267948008
Right trigeminal neuralgia	12242111000119100
Transient arthropathy of the ankle and/or foot	201994008
Right inguinal pain	15629981000119100
Generalized arthritis	202031002
Heel pain	2733002
Ankylosis of hip joint	24273009
Intractable chronic headache following trauma	290441000119105

© 2025 Nahid NA et al. *JAMA Network Open.*

Dislocation of radiocarpal joint	263024002
Crystal arthropathy of wrist	429419004
Articular cartilage disorder of wrist	429052003
Intractable low back pain	29930001000004100
Traumatic dislocation of distal radioulnar joint	733250006
Pain in hallux	418237007
Mallet finger of left hand	313531000119103
Chronic paroxysmal hemicrania	95654002
Loosening of hip joint prosthesis	240267008
Closed traumatic dislocation of metatarsophala...	64438006
Lesion of ligaments of foot region	240029004
Chronic tophaceous gout of knee due to renal i...	303961000119104
Mallet finger of right hand	313521000119101
Closed traumatic dislocation of proximal end o...	73387003
Lesion of ligaments of the ankle region	240019006
Piriformis syndrome	129179000
Chronic pain of left upper limb	15743561000119100
Coxa magna	296041000119103
Closed traumatic dislocation of carpometacarpa...	45634004
Chronic migraine without aura	431601000124105
Paresthesia of hand	309086004
Lower limb joint arthritis	250131003
Open dislocation of ankle	54394007
Primary chronic gout without tophus of wrist	310121000119103
Postherpetic neuralgia	2177002
Numbness of upper limb	298753001
Prepatellar bursitis of right knee	318611000119100
Osteoarthritis of joint of left wrist	318681000119106
Occipital headache	330007
Pain in the coccyx	34789001
Complete tear of ligament of finger	209612008
Pain of right shoulder blade	774134007
Prepatellar bursitis of left knee	318601000119103
Migraine variant with headache	445322004
Gout of shoulder due to renal impairment	298941000119101
Closed traumatic dislocation of sternum	33358005
Closed posterior dislocation of proximal end o...	14741001
Tailor's bunion	7951001
Pain by sensation quality	410720000
Monoarthritis of joint of shoulder region	698477003
Synovitis/tenosynovitis - wrist	287019009
Monoarthritis of hip joint	427947000

© 2025 Nahid NA et al. *JAMA Network Open.*

Ankylosis of the elbow joint	202307002
Closed lateral dislocation of elbow	75824002
Crystal arthropathy of shoulder region	34427002
Stomach ache	271681002
Gouty arthritis of left hand	310201000119103
Pain in right heel	1076791000119100
Arthritis of right wrist caused by bacteria	1073261000119100
Lyme arthritis	33937009
Pain in penis	285375003
Arthritis of right knee	1074861000119100
Headache following lumbar puncture	398987004
Migraine aura without headache	230465000
Arthritis of elbow	439656005
Anterior knee pain	239733006
Mastodynia of right breast	12242511000119100
Mechanical pain	9626006
Closed traumatic dislocation sternoclavicular ...	209117003
Subluxation of foot joint	263061006
Gouty arthritis of toe	428839004
Tear of medial meniscus of knee	302932006
Acute pain in face	735937001
Ankylosis of joint of ankle AND/OR foot	267936005
Neurological pain disorder	247384001
Foreign body sensation	247342009
Elongated styloid process syndrome	609143007
Fibrositis	56557000
Open dislocation of hip	84920009
Facial paresthesia	95665007
Aching headache	162307009
Old posterior cruciate ligament disruption	202111003
Facial neuralgia	4151000119102
Venous intermittent claudication	95443002
Erythromelalgia	37151006
Wound pain	298012000
Acquired left hallux valgus	446321000124103
Acquired right hallux valgus	446271000124106
Osteoarthritis of joint of right wrist	318731000119102
Ankle joint deformity	299418001
Osteoarthritis of joint of left elbow	318651000119104
Osteoarthritis of foot joint	309246000
Effusion of joint of right wrist	306991000119103
Myalgia caused by statin	16462851000119100

© 2025 Nahid NA et al. *JAMA Network Open.*

Complete tear ulnar collateral ligament	209597008
Osteoarthritis of ankle	239874001
Ophthalmic migraine	95655001
Tightness in throat	247323005
Open traumatic dislocation, midtarsal joint	209003005
Total body pain syndrome	279044000
Open traumatic dislocation of tarsometatarsal ...	209004004
Open traumatic dislocation midcarpal joint	208828005
Transient arthropathy-knee	202010000
Closed traumatic dislocation of cervical vertebra	111648001
Traumatic arthropathy of multiple sites	201940003
Traumatic arthropathy of shoulder	201941004
Traumatic arthropathy of the shoulder region	201932009
Arthropathy associated with a hypersensitivity...	53338001
Complex regional pain syndrome, type II, upper...	408750000
Intractable chronic tension headache	290531000119102
Deficiency of ligaments of knee joint	239723003
Congenital syphilitic osteochondritis	58392004
Rheumatoid arthritis of left hand	1073711000119100
Rheumatoid arthritis of left hip	1073721000119100
Rheumatoid arthritis of left knee	1073731000119100
Rheumatoid arthritis of right hip	1073801000119100
Rheumatoid arthritis of right knee	1073811000119100
Congenital dislocation of hip	48334007
Paresthesia of right upper limb	15973701000119100
Paresthesia of right lower limb	15634791000119100
Paresthesia of bilateral hands	840319006
Palindromic rheumatism of shoulder region	202457009
Palindromic rheumatism of pelvic region and thigh	202461003
Palindromic rheumatism of multiple sites	202465007
Palindromic rheumatism	50442003
Right-sided piriformis syndrome	291951000119108
Sacral back pain	61486003
Painful ejaculation	50818007
Derangement of joint of hand	146231000119109
Salmonella arthritis	71299003
Chronic ankle pain	51881000119109
Deficiency of lateral collateral ligament of t...	239730009
Charcot's joint of foot	309255002
Chondromalacia of left shoulder	16039191000119100
Primary chronic gout without tophus of hand	309701000119101
Current tear of lateral cartilage of knee	698558002

© 2025 Nahid NA et al. *JAMA Network Open.*

Kashin-Bek disease	270505009
Chondrocalcinosis due to dicalcium phosphate c...	201625003
Reactive arthropathy of knee	201569000
Chondrocalcinosis of joint of pelvis	445496008
Rebound tenderness of central region	301415004
Daily headache	571000119103
Rebound tenderness of left hypochondrium	301414000
Juvenile osteochondritis of the hip and pelvis	268022000
Juvenile idiopathic arthritis	410502007
Non-traumatic rupture of patellar tendon	202963006
Recurrent dislocation of hand	10464008
Deficiency of anterior cruciate ligament	239724009
Recurrent dislocation of interphalangeal joint	202242000
Post-infective arthritis of joint of hand	423310007
Crystal arthropathy of hand	26241001
Recurrent dislocation of wrist	202230005
Recurrent subluxation of the patella	202248001
Left-sided piriformis syndrome	291941000119106
Refractory migraine variants	425365009
Joint pain in right hand	1076721000119100
Localized, secondary osteoarthritis of the hand	201852006
Scapulalgia	20793008
Bilateral calf pain	15634471000119100
Loosening of knee joint prosthesis	281448003
Benign exertional headache	103011009
Chronic tophaceous gout	73877009
Basilar migraine	83351003
Hypnic headache	122711000119109
Chronic tophaceous gout of hand caused by drug	306171000119102
Pain of left temporomandibular joint	12240271000119100
Pain of left shoulder joint	15916971000119100
Hip pathological dislocation	239759009
Clavicle pain	203509009
Hill-Sachs lesion	202141001
Climacteric arthritis of multiple sites	201985002
Aseptic necrosis of medial femoral condyle	17926002
Pain of bilateral hands	15634551000119100
Articular cartilage disorder of the pelvic reg...	202137000
Congenital absence of hand	371199008
Crystal arthropathy of pelvis	445477009
Complex regional pain syndrome type I of left ...	293931000119101
Lumbosacral nerve root pain	103016004

© 2025 Nahid NA et al. *JAMA Network Open.*

Hemarthrosis of the ankle	202415003
Chronic thoracic back pain	136791000119103
Bilateral headache	162301005
Chronic pain in face	432615008
Bilateral hip joint pain	12247611000119100
Secondary multiple arthrosis	201825001
Secondary osteoarthritis	443524000
Sensory disturbance in limb	395080004
Loose body in ankle joint	202162000
Loose body in elbow joint	202158006
Seropositive rheumatoid arthritis of multiple ...	11055151000119100
Burning feet	36031001
Loose body in joint of ankle and/or foot	267917000
Congenital discoid meniscus	70690000
Slipped upper femoral epiphysis	26460006
Pain of right temporomandibular joint	12240311000119100
Soreness	71393004
Spinal arthritis deformans	53332000
Bouchard's node	20243008
Bone pain	12584003
Loose body in right knee joint	313321000119105
Bilateral wrist pain	11874831000119100
Bilateral shoulder joint pain	12247771000119100
Diffuse pain	2134003
Bilateral sciatica	15633361000119100
Chronic post-thoracotomy pain syndrome	98611000119104
Closed anterior dislocation of hip	111649009

© 2025 Nahid NA et al. *JAMA Network Open.*

eTable 3. Characteristics of the Patients Before and After Inverse Probability of Treatment Weighted (IPTW) Balancing

Characteristics	Before IPTW			After IPTW		
	pNM and pUM	pPM and pIM	SMD	pNM and pUM	pPM and pIM	SMD
	n= 15709 (%)	n= 15960 (%)		n= 31661 (%)	n= 31670 (%)	
**Age ≥45 y**	10216 (65)	10967 (68.7)	0.08	21177 (66.3)	21191 (66.9)	<0.001
**Sex (Females)**	10227 (65.10)	10838 (67.91)	0.06	21051 (66.3)	21054 (66.5)	0.002
**Race**						
White	8747 (55.68)	10570 (66.23)	0.3	19318 (61)	19317 (61)	<0.001
Black	2968 (18.89)	2624 (16.44)		5588 (17.7)	5598 (17.7)	
Other/None indicated	3994 (25.42)	2766 (17.33)		6755 (21.3)	6755 (44.6)	
**Pain diagnoses**						
Back pain	2031 (12.93)	2511 (15.73)	0.08	4520 (14.3)	4522 (14.3)	<0.001
Pain in hand leg joint	3991 (25.41)	4479 (28.06)	0.06	8442 (26.7)	8451 (26.7)	<0.001
Rheumatoid arthritis	3547 (22.58)	4183 (26.21)	0.08	7716 (24.4)	7719 (24.2)	<0.001
Headache(including migraine)	1416 (9.01)	1618 (10.14)	0.04	1990 (9.4)	3008 (9.5)	0.002
Neuropathic pain	1810 (11.52)	2330 (14.60)	0.09	4132(13.1)	4133 (13.0)	0.00
Fibromyalgia	182 (1.16)	333 (2.09)		433 (0.7)	568 (0.9)	0.02
Injury	4033 (25.67)	4220 (26.44)	0.2	8256 (26.1)	8251 (26.0)	<0.001
**Comorbidities**						
Depression	2036 (12.96)	3339 (20.92)	0.21	5352 (16.9)	5365 (16.9)	<0.001
Psychoses	512 (3.26)	798 (5.00)	0.09	1325 (4.2)	1312 (4.1)	0.002
Anxiety	1775 (11.30)	2611 (16.36)	0.15	4375 (13.8)	4376 (13.8)	<0.001
Opioid use disorder	168 (1.07)	216 (1.35)	0.02	394.04 (1.2)	387.19 (1.2)	0.001
Diabetes	2424 (15.43)	2385 (14.94)	0.1	4785 (15.1)	4787 (15.1)	<0.001
Renal failure	927 (5.90)	867 (5.43)	0.02	1809 (5.7)	1800 (5.7)	0.001
Liver disease	846 (5.39)	821 (5.14)	0.01	1663 (5.3)	1658 (5.2)	<0.001
**Medication history**						
Opioid or non-opioid pain med	8581 (54.62)	8637 (54.12)	0.01	17219 (54.4)	17209 (54.3)	0.001
Benzodiazepines and other sedative/hypnotics	4723 (30.07)	5430 (34.02)	0.08	10143 (32.0)	10133 (32.0)	0.001
Antipsychotics	2026 (12.90)	2829 (17.73)	0.13	4866 (15.4)	4860 (15.3)	<0.001
CNS meds/ stimulants	168 (1.07)	414 (2.59)	0.13	5640 (17.8)	5615 (17.7)	0.002
Skeletal muscle relaxants	3170 (20.18)	3613 (22.64)	0.06	6784 (21.4)	6769 (21.4)	0.001
**MME/ day**						
<20	2969 (18.9)	3114 (19.5)	0.06	6087 (19.2)	6083 (19.2)	<0.001
20- <30	5585 (35.5)	5925 (37.1)		11457 (36.2)	11469 (36.2)	
≥30	7155 (45.5)	6921 (43.4)		14119 (44.6)	14120 (44.6)	
**ED Visits (last six mo)**	2638 (16.8)	2707 (16.9)	0.003	5348	5341	<0.001

pUM: phenotypic ultra metabolizer; pNM: phenotypic normal metabolizer; pIM: phenotypic intermediate metabolizer; pPM: phenotypic poor metabolizer. pUM, pNM, pIM, pPM were based on genetic activity and concomitant use of CYP2D6 inhibitors; SMD: Standardized Mean Difference

© 2025 Nahid NA et al. *JAMA Network Open.*

eTable 4. Distribution of the Different Medications in the Study Population

Prescriptions	pNM and pUM	pPM and pIM
	**n= 15709 (%)**	**n= 15960 (%)**
Opioids		
Hydrocodone	3960 (25.21)	4226 (26.48)
Tramadol	2769 (17.63)	2968 (18.60)
Codeine	2826 (17.99)	2725 (17.07))
Oxycodone	6154 (39.17)	6041 (37.85)
Strong Inhibitors		
Bupropion	0	1721 (30.43)
Fluoxetine	0	967 (17.10)
Paroxetine	0	367 (6.49)
Terbinafine	0	112 (1.98)
Quinidine	0	2 (0.04)
Moderate Inhibitors		
Duloxetine	37 (2.67)	1396 (24.69)
Mirabegron	2 (0.14)	101 (1.79)
Cinacalcet	1 (0.07)	42 (0.74)
Lorcaserin	1 (0.07)	13 (0.23)
Abiraterone	0	0
Rolapitant	0	0

© 2025 Nahid NA et al. *JAMA Network Open.*
