# Supplementary material for: CYP2D6 Phenotypes and Emergency Department Visits Among Patients Receiving Opioid Treatment
Source: JAMA Netw Open. 2025 Jul 28;8(7):e2523543. doi: 10.1001/jamanetworkopen.2025.23543 (PMC12305384; doi:10.1001/jamanetworkopen.2025.23543)
Supplement: Supplement 2. — Data Sharing Statement [file jamanetwopen-e2523543-s002.pdf]

Data Sharing Statement

Nahid. CYP2D6 Phenotypes and Emergency Department Visits Among Patients Receiving Opioid Treatment. *JAMA Netw Open*. Published July 28, 2025.
doi:10.1001/jamanetworkopen.2025.23543

Data
Data available: No

Additional Information
Explanation for why data not available: All of Us Research Program data is broadly available via the All of Us Research Program Researcher Workbench to registered users.
